# Indents

**Published:** 1917-08

**Authors:** 


### INDENTS

**☞ Brief Articles of Technical or Scientific Value will receive notice and comment. Contributions invited.**

*The Error of Condensing Amalgam Fillings*

Of late years there has been a tendency to regard amalgam as a substance to be manipulated in much the same manner as is gold foil—that is, to strongly compress it as it is being packed into the tooth cavity; then, when the filling has been built up to its required dimensions, to use a very large, broad-faced amalgam plugger that will cover as much as possible of the outer surface of the filling, and by the application of all of the energy that the filling will stand, force the plugger down and so ''condense'' the filling.

In order to properly appraise the value of such a procedure, it is only necessary to compare the physical characteristics of the two widely different materials, gold foil and amalgam. In gold foil we have a substance that is in a state of relaxation and is inherently soft, and therefore capable of being compressed under a plugger as each successive layer is placed in position. In the unappropriated alloy content of dental amalgam, however, we have a substance that can not be compressed by any force that the dentist can exert on a filling.

Thus, while gold will flow under a comparatively insignificant pressure, a recent test of the amalgam alloy, Ag2SnCu developed a resistance of over 120,000 pounds to the square inch, truly a compressive strength that even a dentist can not hope to overcome with only his ''good right arm'' and a broad-faced plugger. Pure gold foil unites or welds under pressure as the result of the force of molecular cohesion, this becoming operative through the transmission of external energy. An amalgam, on the contrary, unites as the result of the operation of the force of crystalline adhesion, which latter force is operative only after the molecules of amalgam or other alloys have been built up into the form of crystals, as the result of the operation of certain forces of chemical and physical attraction.

Crystalline adhesion requires contact of the planes of the crystals, but strong compression is not necessary to secure this contact, for the force of crystalline adhesion is automatically exercised where the amalgam paste has been spatulated or burnished into place in the tooth cavity as a filling. Gold, after compression, is in a state of comparative equilibrium of volume, which is only disturbed by such thermal changes as occur in the human mouth, and moreover, there are no chemical reactions occurring within it nor such a physical phenomenon as diffusion. Amalgam, on the contrary, is not in volume equilibrium until its mercury content has, by osmotic pressure, diffused through the mass and its every particle has gone into chemical combination with the molecules of the contained alloy, thus forming crystals of definite composition and fixed physical properties.

From this it can be seen that however strongly an amalgam filling has been compressed, it is certain that the hoped-for condensation, which in effect has resulted in setting up internal strains and in driving a portion of the mercury to the margins of the filling, will be completely nullified by the internal motion caused by the operation of natural laws which govern all the phenomena of alloying. —W. W. Atkinson, *N. J. Dental Journal.*

*Royal Dentistry in Morocco*

Now and again a dentist is driven to fight for his just reward by setting forth in detail the operations that led to the presentation of his "memorandum of his fee for professional attendance" and insisting on his tale of guineas if necessary in the equivocal atmosphere of a court of law. But it does not often happen that the defendant is a monarch, whether uneasily seated on a shaky throne or retiring into private life on a state pension. Monarchs are growing scarcer and scarcer, very soon will be the isolated dentist pursuing single-handed his romantic career. The dentistry of the future will plod in full daylight along the ordered garden paths of public service. These facts heighten the interest of the story of the experiences of the court dentist of the late Sultan of Morocco as recently recounted by the Morocco correspondent of the *Times*, who was called in by both parties to mediate between the Sultan and the French government as to the payment of the retiring monarch's debts. These debts included some very long bills. One was for a marble staircase ordered in Italy for the palace at Fez. The representatives of the French government argued that the staircase was a piece of extravagance and that the Sultan ought to pay for it himself. The Sultan pointed out that the state stood to benefit by the improvement to its property and in the end the French Protectorate paid the bill. When it was paid the Sultan telegraphed privately to Italy and had the staircase landed and set up at the new palace he was building for himself at Tangier. There was also a bill for several hundreds of yards of crimson cloth for trousers for the women servants of the Imperial kitchens. This the Sultan fought on the ground that the kitchen slaves were part of the Imperial entourage and their clothing as much a state debt as the uniforms of the soldiery. The Protectorate authorities complained of the unnecessary richness of the material. "It may be the custom in Europe," replied the ex-Sultan, "for the royal kitchenmaids to wear cotton trousers, but in Morocco we have more sense of the dignity of their position." This argument silenced the Protectorate and the bill was paid.

But the debt whose settlement caused the greatest amount of trouble was the dentist's bill. This bill was not what one might naturally suppose it to be. It had no relation to work done in the mouths of the Sultan and his household. It was for a live lion. It seems that the Sultan, when he had successfully usurped the throne of his brother, had immediately begun purchasing wild animals on a large scale. He sent his dentist to Berlin to visit Hagenbeck's collection and to buy suitable specimens. This commission the dentist carried out, but he lingered on in Berlin for some months after the despatching of the menagerie, and on his return to Morocco found that the Sultan's fancy had passed, and that there was a lion that had never been paid for. He was personally answerable to Hagenbeck and plied the Sultan for the price of the beast. The Sultan's defense was an acknowledgment of the debt and a counterclaim against the dentist for a throne constructed to go up and down like a dentist's chair. This throne long promised by the dentist had never been delivered. The Sultan also said that the bill for the lion was not a personal, but a state debt and no concern of his, and that he would have nothing to do with it, that moreover he had paid it and if he had not paid it it was a state debt. He further claimed to have paid for the mechanical throne.

When the hostilities were at their height the Sultan ordered the dentist to give up his residence, a small villa on one of the Sultan's properties in Tangier. The dentist refused and a body of slaves was sent down to evict him. They were met with barricades and pistol shots. At this point the *Times* correspondent was asked by the French Protectorate to arrange a meeting between the parties and endeavor to bring them to terms. He interviewed them both separately and it was agreed that the dentist should be paid a certain sum and that there should be a reconciliation; after which all claims should be dropped. The result of the interview was that the dentist, who was a Spaniard, was removed, struggling and shouting his demands, from the presence of the Potentate who, almost held down on his throne by the peacemaker, pursued the dentist with violent reiterations of his grievance with regard to the chair-throne. The final solution was the taking over of the responsibility for the lion by the French government, who were unaware that meantime it had died. The Sultan never got his mechanical throne.—*Dental Record.*

*Gold or Aluminum Lining for Vulcanite Dentures* When the wax base plate has been removed from mould just before packing in the rubber, coat model with liquid silex and immediately sift on some powdered aluminum or powdered gold as you may desire. Now dissolve a piece of same kind of rubber used for plate in chloroform and add double the amount of powdered aluminum to the bulk of the solution and by means of a camel's hair brush coat the model with same.

Allow sufficient time for the chloroform to evaporate before packing rubber and closing flask.

A lining prepared in this way is durable and can not possibly pull off. The lining prevents any action of silex on the plate in vulcanizing, and the silex prevents the plaster from sticking to the palatal portion of the plate.—T. W. Crozier, D. D. S., Christiansburg, Va.

*Abolish Liquor, Doctors Urge* New York, June 4.—Prohibition for the period of the war was advocated tonight at a meeting held under the auspices of the American Medical Association's committee on the treatment of alcoholism and narcotic addiction. The sense of the meeting was that the government not only should prohibit the manufacture of whisky, but that it also should pass laws stopping the use of such as is already manufactured.

Dr. John D. Quackenbos, widely known as an alienist, declared that if the nation is to maintain its normal standing tobacco also must be abolished. He added that the time would come "when the man who deals in drink, cigarettes, or cigars would be deemed a murderer."

*Magazine Postage* The daily press is very much concerned in the proposed increase in second-class mail rates, which will result in an additional revenue of $19,000,000. This has been called a tax on the press.

Mr. Curtis, of the Curtis Publishing Co., Philadelphia, says: "If Von Tirpitz himself had drawn this revenue bill, it could not declare more ruthless warfare on the periodicals of the country."

Our magazines and newspapers, under present conditions, are paying one cent a pound. This is much less than the expense to the government, and the second-class mail rate is responsible for the annual postal deficit. Outside of the desirability of raising money to increase the war revenues, it would seem to be only just that this business pay at least its cost of carrying.

Since the beginning, *Oral Hygiene* has been refused second-class mail rates, as it has no subscription list. For every copy sent through the mails, the publishers pay two cents a copy or at the rate of eight cents a pound, necessitating an expenditure of over $10,000 per annum for postage alone. No one could deny that *Oral Hygiene* is educational and is as much entitled to consideration as most of the publications now enjoying this privilege. It is expected that if this new law goes into effect, many publications will be discontinued. In view of the fact that *Oral Hygiene* has survived under a $10,000 annual tax, it would seem that it is possible for a publication of national circulation to continue its existence with a profit, provided good business management and economics are exercised in its output.

So long as *Oral Hygiene* continues to exist and is penalized by paying a rate eight times in excess of the more favored publications, such arguments are not convincing. —*Oral Hygiene.*

*To Remove Model from Articulator* Use separating fluid on contact side of model, allowing plaster to come up on edge just enough to hold. When ready to remove use sharp knife around edge and model will drop off.—T. A. Rose, in *Digest.*

*Amalgamated Gold* Any cohesive gold foil may be selected and a few pellets placed in a clean mortar and enough mercury added to insure amalgamation without using the pestle. After all pellets have been amalgamated the mass is poured into a chamois bag and the excess removed by squeezing with a pair of pliers. In making small cervical inlays or simple occlusal inlays, forty-four gauge pure gold is burnished into the cavity, special care being taken to see that the gold is properly adapted to the margin. This matrix is then filled one-third full of the amalgamated gold and removed from the cavity. It is then placed on a heater with a very small flame and the mercury very slowly volatilized, great care being used not to drive off the mercury too fast. When the mass regains its original gold color, the matrix may be placed back in the cavity, reburnished to overcome the slight shrinkage and more amalgamated gold added. This procedure is repeated until the cavity is filled and then the inlay can be polished and set.

If for any reason the margin of a cast gold inlay is defective, the inlay can be placed in the cavity and amalgamated gold can be burnished into the space between the inlay and the cavity margin and the entire mass removed and heated very slowly. If marked shrinkage occurs, more amalgamated gold can be added. Should the added part of the inlay be subjected to the strain of mastication, it should be reinforced by solder. Where pits are found after solder operations they should be thoroughly cleansed with a bur and filled with amalgamated gold. The appliance, although it may contain facings, can be very slowly heated and the mercury volatilized. Amalgamated gold may be employed in the repair of gold crowns, making contact points and repairing solder joint Richmond crowns where the solder failed to reach the labial. None of this amalgamated gold need be wasted, for should any remain, more mercury can be added and reamalgamation takes place.—Dr. R. C. Harkrader, *The Dental Summary.*

*Patriotic Work for those Unable to Accompany Dental Corps* The Government wishes to cause as little hardship and sacrifice to the Reserve Dental Corps as may be consistent with the needs of the country. With this end in view we would bring to your attention the necessity of the local unit of the League or in its absence the city, district or state dental society; organizing for the purpose of taking care of the practice of the officers of the reserve, who respond to the call for service.

For example, should Dr. Doe be called to the colors, the local unit through its members, by some equitable plan which would vary in different localities, take care of his practice during his absence. Upon his relief from active duty his practice would be returned to him intact, or as nearly so as possible. Such a plan would cause no unnecessary hardship to the officers responding to the call for service; while the absence of such a plan would penalize the officer who gives his service to the country in a crisis. The League appeals to the patriotism of the profession to protect the interests of the officers and their families.

ONE PLAN

The officer called on duty will mail to his patients a list of League members who have promised to work for them under some equitable plan—possibly turning over part of the fee to his family and upon his return sending all patients back.

ANOTHER PLAN

Volunteer service by League members, who will go to the office on certain days, of the one called away; thus looking after his practice and giving an income (even though small) to the family.

Many other plans will suggest themselves and will be applied to cases when indicated.

*New Law* Indiana has a new dental law which was signed and approved March 9, 1917. It provides for an annual license which is to be renewed each year at an expense of one dollar. The dentist is exempted from jury duty; a Board of Examiners consisting of five reputable practicing dentists, appointed jointly by the governor and the State Dental Association, are features of the new bill.

Reciprocal relations have been established with Ohio, Michigan, Illinois, Minnesota, Iowa, Nebraska, Kansas, Kentucky, Missouri and Louisiana, upon the following terms: "Any applicant who has been in legal and ethical practice in any of the above states for not less than five years and who is a member of the State Dental Society, and has the recommendation of the board and society of his state, may be admitted to our examinations at any regular meeting and excused from all theoretical examination, being required to pass the practical examination only."—*Oral Hygiene.*

*An Esthetic Root Protection* When crowning anterior teeth it very frequently happens that the patient will have to do without that unit of the arch. This is very annoying to those who have to appear in public minus the tooth or crown. The difficulty may be overcome by the use of a Vulcolox tooth. Select one that in size and color matches the case, pass a wire once or twice around the pin and twist the two ends until the attachment is firm. Cut the improvised post to the desired length set, set the crown with temporary stopping. This method serves three purposes: It protects the tissues against the impact of the food, keeps the investing tissues from folding over the root, thus keeping it in full view for the seating of the permanent crown, and is of esthetic value to the patient who is very grateful for this little consideration.—Dr. Fraham, in *Pacific Gazette.*

*Optical Glass* Previous to the European war, the American manufacture of optical instruments was dependent on crown glass of German manufacture. It is stated that four plants in the United States, including the Bausch & Lomb Co., of Rochester, have succeeded in producing a clear optical glass in quantities sufficient to meet commercial demands. This discovery is most timely as it is used in making all kinds of war supplies, including field glasses, range finders, periscopes, telescopes, surveying apparatus, chemical laboratory equipment, and other materials necessary in modern warfare—*Oral Hygiene.*

*Mouth Sepsis* Considerable attention has been bestowed on oral sepsis as an essential etiologic factor in systemic infections, and there is reliable scientific evidence in support of the tooth-root theory of this large class of diseases. The fact that chronic septic foci in teeth and elsewhere are exceedingly difficult to recognize in the majority of cases is undoubted. It is particularly instances of periapical infection or abscess that offer the greatest difficulty in this respect. Here, even a Roentgen examination, which should always be made by an expert, may fail to render reliable aid.

Daland states that the diagnosis of mouth sepsis should be made by a dentist who is specially trained for this, in which opinion I concur. The physician, however, who encounters a case of systemic infection in which the teeth are suspected should refer the patient to a competent nose and throat specialist with a view to eliminating all foci other than those which may be present in the mouth before invoking the services of the specially trained dentist. It sometimes happens that multiple foci are discoverable, in as many different organs, as the teeth, tonsils and sinuses.

Successful treatment of secondary systemic infection demands, first and foremost, the removal of the septic focus or foci on which they depend. Before reducing the masticating surface of a particular patient, however, the evidences of an existing necessity for so doing should be as clear and convincing as possible. An attempt by a competent dentist should be made to heal the morbid lesions in and about the tooth roots, since some of these are amenable to expert management, before ordering the extraction of the teeth.

Within the past six months a number of dentists have informed me that countless teeth are being removed without justification, on the advice of physicians, usually following a Roentgen examination (by amateurs in many instances). In well authenticated cases in which one or two teeth were the seat of peripheral infection, physicians have gone so far as to give emphatic directions to the effect that all of the remaining teeth be extracted. For example, one of Philadelphia's best known specialists in extraction was requested by a physician to pull out all of a certain patient's teeth—twenty—but he courteously, though firmly, declined to do so.

It seems to me that the rapidly growing custom of sacrificing teeth, many of which are merely suspected of being septic, can not fail to arouse the most ardent activity of dentists in opposition thereto, and must prove the ultimate chagrin of the medical profession. It would appear that an amazingly low estimate is being placed on the value of human teeth by an increasing number of physicians, who should appreciate the importance of a good masticating apparatus to the digestive function—to the maintenance of health.

I do not mean to disparage the significance of these latent chronic septic foci as a cause of secondary systemic infections or to depreciate investigations in this, comparatively speaking, new field of endeavor. The object of this letter is to utter a word of warning with a view to lessening what I believe to be an unwarrantable present-day sacrifice of the masticating surface, and to spare the medical profession the adverse criticism of the future, by a broader conception proper to the subject of oral sepsis and its management in the present.—Dr. J. M. Anders, in *Journal of A. M. A.*

*Edward C. Kirk Accepts Directorship in the S. S. White Dental Manufacturing Company*

It is with regret that *The Journal* records that Dr. Edward C. Kirk has given up his professional activities and will hereafter devote his time to business matters. This is evidenced by the fact that he recently resigned from membership in the National Dental Association and severed all teaching relations with the Dental Department of the University of Pennsylvania, and has accepted a directorship in the S. S. White Dental Manufacturing Company, beginning July 1, 1917.

For years Dr. Kirk has been active in all matters pertaining to the advancement of the dental profession. As dean of the Dental Department of the University of Pennsylvania, he has kept himself abreast of dental educational affairs and as editor of the *Dental Cosmos*, he has not only given the profession one of the best journals, but through its pages and by his timely and scholarly editorials he has endeavored to keep the profession in touch with the latest advance and best there was in dentistry. Dr. Kirk's writings have been largely upon educational and scientific subjects and few men in the profession have had a clearer vision of what dentistry should be than this broad thinker. His ideals have ever been high and by his work and writings he has endeavored to bring the profession up to a high standard. Truly a man with such a brilliant mind and such a powerful factor in the profession will be missed from our councils.

Dr. Kirk has also resigned his position as chairman of the Dental Committee and from membership on the General Medical Board of the Council of National Defense. However, the profession is to be congratulated and will be pleased to learn that the General Medical Board, through its Chairman, Dr. Franklin Martin, has appointed to this important position Dr. William H. G. Logan, of Chicago, who was the other dental representative on the General Medical Board. In this crisis it is indeed fortunate that a man of Dr. Logan's proven executive ability was selected to assist in caring for the dental needs of the army and navy and the best interests of the dental profession.—Editorial, in *Journal of N. D. A.*, for July.

*Principal Defects Found in Persons Examined for Service in the United States Navy*

A third and most important cause for rejections is defective teeth, which in itself is surprising when one takes into consideration the number of dentists in this country. Nevertheless, twenty-seven per cent. of the rejections at the recruiting stations are for teeth which have been neglected until their condition is such that they are beyond possibility of repair. The minimum requirements of twenty sound teeth, of which there must be four opposing molars and four opposing incisors, with crown and bridgework counting as sound teeth, are fair, and a man's mouth could hardly be considered in good condition without conforming to this requirement. A peculiar coincidence I have noticed in the examination of applicants who have defective teeth is the frequency that defective vision is present in the same individual; from this I am led to believe that the man, with a mouthful of decayed teeth, develops a toxin which in some way is partially responsible for the condition of his eyes. Teeth, as we now understand them, are the cause of many of our ills, and as it is necessary for a man in the navy to be in a constant state of good health his teeth must be in a good condition, otherwise, he is a victim of his own toxins; and living, as he necessarily has to, in confined quarters aboard ship he is less resistant to outside infections, as well as a menace to other members of the crew, through common use of the same drinking fountains and as a bacteria carrier. Men of this character are not desirable and must be rejected temporarily until they have the proper dental work done.—Dr. Costello, in *The American Journal of Public Health.*

